# A simple and rapid vascular anastomosis for emergency surgery: a technical case report

**DOI:** 10.1186/1749-7922-4-30

**Published:** 2009-08-03

**Authors:** Chad G Ball, David V Feliciano

**Affiliations:** 1 Department of Surgery, University of Calgary, Foothills Medical Center, 1403-29 Street, Calgary, Alberta, Canada, T2N 2T9, USA; 2 Department of Surgery, Emory University, Grady Memorial Hospital, 69 Jesse Hill Jr Drive, Glenn Memorial Building, Suite 302, Atlanta, Georgia, USA, 30303, USA

## Abstract

A 22-year old male presented with a transected femoral artery following a gunshot wound. He underwent a successful primary repair following limited segmental resection of the injured segment. End-to-end anastomoses after resection of injured arteries include, but are not limited to, interrupted and continuous suturing with, or without "parachuting" of the graft and/or vessel. We offer a rapid and reliable repair using a conceptually and operationally simple technique. Major advantages include: 1) the operating system is always oriented towards the surgeon, 2) the posterior row of sutures is placed as both ends are readily visualized, avoiding the need for potentially obscuring traction stitches, and 3) flushing is easily performed prior to completing the anterior suture row.

## Background

End-to-end anastomoses after resection of injured arteries were described in the United States as early as 1897, however it was not until the later stages of World War II, and then the Korean War that they became an acceptable solution for the management of acute vascular injuries [1-4]. Although Carrel and Guthrie are credited with describing an end-to-end anastomosis using triangulation with 3 equidistant sutures [5], other techniques have since been published [6]. These include, but are not limited to, interrupted and continuous suturing with, or without "parachuting" of the graft and/or vessel [6]. A simple and rapid method for end-to-end anastomosis after limited segmental resection of an injured femoral artery is described in this report.

## Case presentation

A 22-year old, otherwise healthy, male presented following a single gunshot wound to the left groin. On examination, the patient was hemodynamically stable, but had no palpable lower extremity pulses on the injured side (dorsalis pedis or posterior tibial). The ankle-brachial index confirmed an arterial injury (<0.9). On immediate exploration, a transacted superficial left femoral artery was identified. Following debridement of the contused ends of the vessel, as well as moderate mobilization, a primary repair was completed using the technique described. The patient was discharged home on post-operative day 3 with normal extremity function.

## Discussion of technique

As with most vascular anastomoses, a synthetic, nonabsorbable monofilament suture on an atraumatic needle (6-0 polypropylene) was employed. Basic principles of vascular repair were followed. These included debridement of contused or lacerated vessel, proper orientation, and an absence of tension on the anastomosis. We did not require an autalogous graft (reversed saphenous vein).

**Figure 1:**
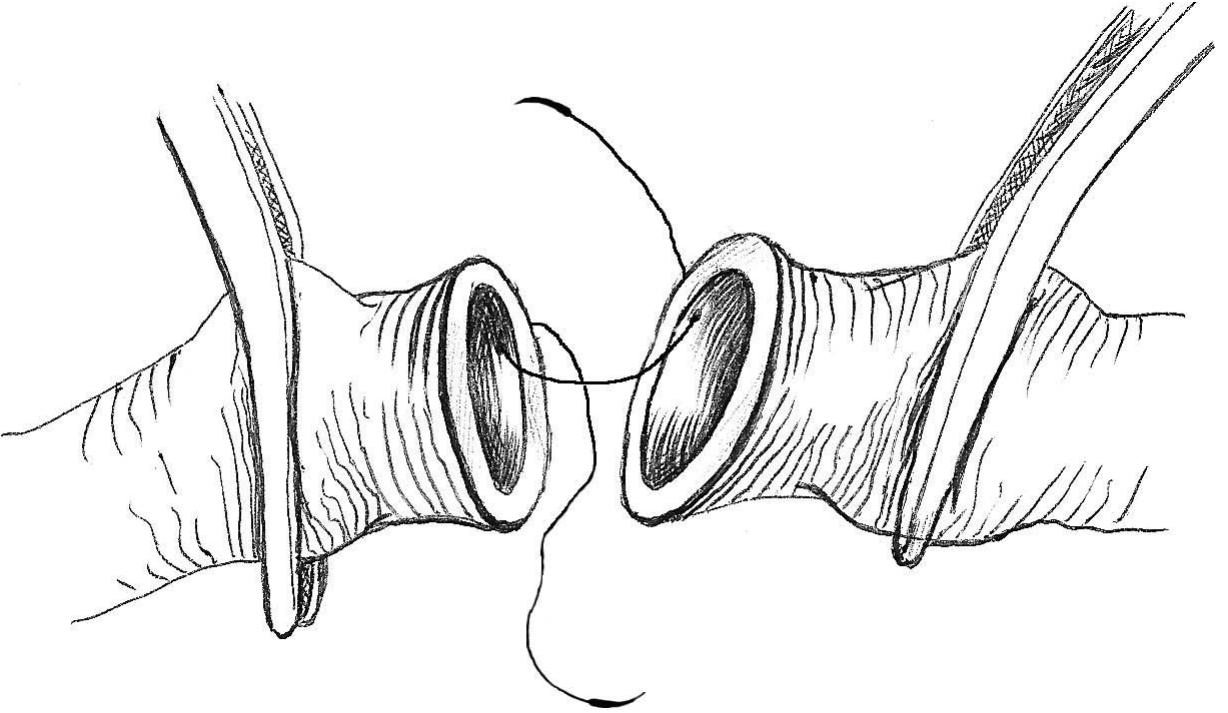
**Vascular anastomosis beginning at the position opposite the operator**.

**Figure 2:**
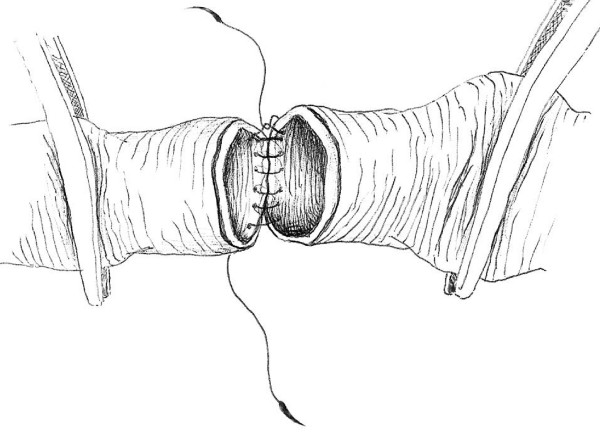
**Completed posterior wall suture line**.

**Figure 3:**
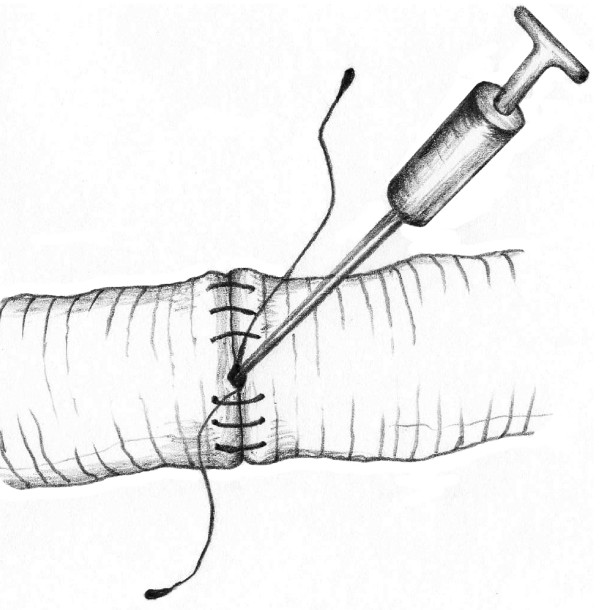
**Flushing the vessel with heparinized saline**.

**Figure 4:**
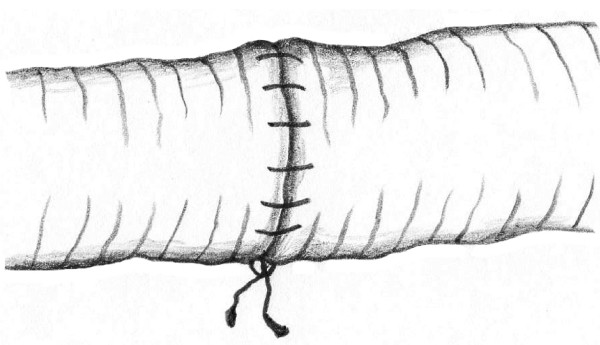
**Completed anastomosis with knot on operator's side**.

This technique of vascular anastomosis requires a double-armed polypropylene suture placed in a continuous fashion with perpendicular bites located 1 mm from the vessel edge and 1 mm apart. The anastomosis begins at the position opposite the operator (3 or 9 o'clock depending on the patient side) where the first 2 bites are placed from inside to outside the vessel using both arms of the suture (Fig. 1). After tying these 2 suture ends together, one end is passed from outside to inside on the posterior aspect of the vessel (adjacent to the first knot of the proximal end) (Fig. 2). The posterior suture line is typically completed first, followed by the anterior side (Fig. 2). Prior to completing the last few bites of the anterior row, the vessel is flushed of debris and air using sequential distal and proximal clamp releases in the standard fashion. After reapplication of the vascular clamps, the visible lumen is flushed with heparinized saline, and the last few bites of the anterior row are completed (Figs. 3 &4). To eliminate air from the system, the distal vascular clamp is removed before the final knot is tied at the 3 or 9 o'clock position. Restoration of pulses at the wrist after end-to-end anastomosis of the subclavian, axillary, or brachial artery is considered excellent evidence of a satisfactory repair in the upper extremity. With end-to-end anastomosis of the iliac, popliteal, or tibioperoneal artery after trauma, completion arteriography is preferred to differentiate the presence of vascular spasm from distal in situ thrombosis or distal embolization into the popliteal or shank arteries.

## Conclusion

Although techniques of vascular anastomosis after trauma are numerous in type and form, most surgeons will default to the one associated with the greatest comfort and ease. This report offers a rapid and reliable repair using a conceptually and operationally simple technique. Its methodology is appropriate for all repairs, including cases mandating the insertion of vascular conduit. We have employed this technique for the past 15 years in nearly all patients with vascular injuries, regardless of the site and size of the vessel. This has included vessels of the neck, torso, upper and lower extremities. There have been no obvious complications associated with its use. Major advantages include: 1) the operating system is always oriented towards the surgeon, 2) the posterior row of sutures is placed as both ends are readily visualized, avoiding the need for potentially obscuring traction stitches, and 3) flushing is easily performed prior to completing the anterior suture row.

## Competing interests

The authors declare that they have no competing interests.

## Authors' contributions

Both CGB and DVF conceived, wrote, and edited the manuscript. Accompanying images were conceptualized by DVF, and completed by a professional biomedical artist. Both authors read and approved the final manuscript.

## Consent

Written informed consent was obtained from the injured patient for publication of this case report. A copy of the written consent is available for review by the Editor-in-Chief of this journal.
